# COVID-19 turned upside down: A psychological perspective

**DOI:** 10.1192/j.eurpsy.2021.1798

**Published:** 2021-08-13

**Authors:** V. Gairola

**Affiliations:** School Of Human Studies, Ambedkar University Delhi, Delhi, India

**Keywords:** COVID-19, psychiatry, Psychology, Psychoanalysis

## Abstract

**Introduction:**

Only little is known about COVID-19 which is now playing its engulfing function in terms of devouring global health, resulting in a crisis that is as novel as the novel Coronavirus strain itself. Both, the structure and function of COVID-19 have been documented and further research is in progress to fill the lacuna. With significant levels of globalization, COVID-19 spreads rapidly around the globe.

**Objectives:**

Masses are hoping for a vaccine as their ultimate object of liberation. People are talking about crashing economy, but what about the crash of humans from their being? Here, a biological, psychological, and psychoanalytic understanding of COVID-19 is investigated. The impact of physical isolation has been documented, but mental isolation remains an uncharted space. The psychological trace—the paleontological psychic trauma of experiencing a pandemic as a witnessing subject is not much talked about. This effort is to open the paths to understanding COVID-19 which may seem pathless at first.

**Methods:**

Primary sources are used along with an intradisciplinary and interdisciplinary unison of psychiatry, psychology, psychoanalysis, yoga, and meditation.

**Results:**

Yoga and meditation for strengthening both physical and psychological immunity along with facilitating the acceptance of psychological impact which is unregistered in the minds of a large population is elucidated.

**Conclusions:**

It is in the interdisciplinary and intradisciplinary unison that preparedness for future pandemics could be curated.

**Disclosure:**

No significant relationships.

